# Identification of functional features of synthetic SINEUPs, antisense lncRNAs that specifically enhance protein translation

**DOI:** 10.1371/journal.pone.0183229

**Published:** 2018-02-07

**Authors:** Hazuki Takahashi, Ana Kozhuharova, Harshita Sharma, Masakazu Hirose, Takako Ohyama, Francesca Fasolo, Toshio Yamazaki, Diego Cotella, Claudio Santoro, Silvia Zucchelli, Stefano Gustincich, Piero Carninci

**Affiliations:** 1 RIKEN Center for Life Science Technologies, Division of Genomic Technologies, Yokohama, Kanagawa, Japan; 2 RIKEN Center for Life Science Technologies, Division of Structural and Synthetic Biology, Yokohama, Kanagawa, Japan; 3 Area of Neuroscience, Scuola Internazionale Superiore di Studi Avanzati, Trieste, Italy; 4 Department of Health Sciences, Università del Piemonte Orientale, Novara, Italy; 5 Department of Health Sciences & IRCAD, Università del Piemonte Orientale, Novara, Italy; 6 Department of Neuroscience and Brain Technologies, Istituto Italiano di Tecnologia, Genova, Italy; 7 TransSINE Technologies, Yokohama, Kanagawa, Japan; John Curtin School of Medical Research, AUSTRALIA

## Abstract

SINEUPs are antisense long noncoding RNAs, in which an embedded SINE B2 element UP-regulates translation of partially overlapping target sense mRNAs. SINEUPs contain two functional domains. First, the binding domain (BD) is located in the region antisense to the target, providing specific targeting to the overlapping mRNA. Second, the inverted SINE B2 represents the effector domain (ED) and enhances translation. To adapt SINEUP technology to a broader number of targets, we took advantage of a high-throughput, semi-automated imaging system to optimize synthetic SINEUP BD and ED design in HEK293T cell lines. Using SINEUP-GFP as a model SINEUP, we extensively screened variants of the BD to map features needed for optimal design. We found that most active SINEUPs overlap an AUG-Kozak sequence. Moreover, we report our screening of the inverted SINE B2 sequence to identify active sub-domains and map the length of the minimal active ED. Our synthetic SINEUP-GFP screening of both BDs and EDs constitutes a broad test with flexible applications to any target gene of interest.

## Introduction

One of the key conclusions of the FANTOM project is that the majority of the genome is transcribed and the majority of transcripts are constituted by long non-coding RNAs (lncRNAs) [1]. Additionally, a substantial portion of lncRNA sequences are antisense to protein coding mRNAs, forming sense-antisense (S/AS) pairs [2]. S/AS pairs are very abundant, involving at least 72% of all genome-mapped transcriptional units identified in the mouse transcriptome in the FANTOM3 project [2]. Various types of regulatory functions have been generally assigned to lncRNAs associated with S/AS pairs. Antisense RNAs may positively regulate sense mRNA transcription, like in the case of the antisense transcript of β-secretase-1 (*BASE1*-AS). This lncRNA has been pathophysiologically associated with Alzheimer’s disease and up-regulates transcription of the sense transcript [3]. On the other hand, antisense RNAs may negatively control levels of transcription, as in the cases of the lncRNAs located antisense to the brain-derived neurotrophic factor (BDNF), the glial-derived neurotrophic factor and the ephrin receptor B2 mRNAs [4].

We recently identified a novel functional class of antisense lncRNA, which acts to positively regulate translation of partially overlapping protein-coding mRNAs [5]. The representative members of this new functional class are the AS Uchl-1, a lncRNA antisense to the ubiquitin carboxyl-terminal hydrolase L1 (Uchl-1) and the AS Uxt, the antisense transcript of the ubiquitously expressed transcript (Uxt). While Uchl-1 is involved in brain function and neurodegeneration [6], Uxt plays a role in tumorigenesis [7, 8]. Both AS Uchl1 and AS Uxt overlap sense mRNAs at the 5’ ends in a “head-to-head” divergent configuration. We discovered that the short interspersed nuclear element B2 (SINE B2) sequence, embedded in an inverted orientation, is required to enhance the translation of the respective sense mRNAs (Uchl1 and Uxt) [5]. We named this new functional class of antisense transcripts “SINEUPs”, as they require a SINE sequence to UP-regulate translation of sense mRNA in a sequence-specific manner [9–12]. Interestingly, we could transfer this property to other mRNAs by simply modifying the antisense region, which we termed the binding domain (BD). Accordingly, the BD of AS-Uchl-1 was modified to target Enhanced Green Fluorescent Protein (EGFP) by substituting the antisense sequence so that it would hybridize with EGFP mRNA. This construct, named SINEUP-GFP, was found to enhance translation of EGFP in HEK293T cell lines and various other mammalian cell lines [5].

SINEB2 are retrotransposable elements, which share evolutionally ancestors with tRNAs [13]. During genome evolution, SINEs have been randomly retrotransposed in a multitude of locations along the mouse chromosomes [14]. SINEB2 elements contain two RNA polymerase III promoter elements, the A box and the B box, and a RNA polymerase II (RNA pol II) binding region [15–17]. Historically ncRNAs, including repetitive elements (REs), were believed to be junk or parasitic remnants of the genome; however, more recently, we and others have unexpectedly identified various biological functions of REs. For instance, in the ES and iPS cells, in cancer and in brain, RE elements have been found to be actively transcribed and provided by regulatory elements, such as enhancers and promoters, to maintain these cells in their undifferentiated status [18–20]. SINE B2 RNAs are also known as functional ncRNAs that directly bind to RNA pol II to repress transcription after heat shock in mouse cells [16, 21]. These results suggest that these elements have been adapted to regulate cellular responses. In the case of SINEUPs, SINE elements represent embedded functional domains that provide the translation enhancer function to antisense lncRNAs. This domain organization has been proven for mouse-derived SINE B2 repeats [5, 9, 10] and for partial Alu and MIR elements in human antisense lncRNAs [22].

In mouse cells, when Uchl-1 translation is enhanced by its antisense upon cell stress with rapamycin, Uchl-1 mRNAs were observed to be associated with actively translating polyribosomes compared to the control (non-stressed) conditions [5]. Additionally, EGFP-mRNA association with polyribosomes was increased by synthetic SINEUP activity [23]. Various reports found that stress, such as viral infection and heat shock, induces and accumulates human Alu RNA and mouse SINE B1 and B2 transcription. For instance, human Alu non-specifically regulates translation initiation *in vitro* [24–29], with mechanisms that likely differ from SINEUP-mediated translation enhancement. Double stranded RNA (dsRNA) produced during viral infections and interferon response cause activation of dsRNA-dependent protein kinase (PKR) by its auto-phosphorylation. Consequently, eukaryotic translation initiation factor 2A (EIF-2a) is also phosphorylated, interfering with translation initiation [30]. When human Alu RNAs are accumulated *in vitro*, PKR activity is thought to be regulated by human Alu binding to PKR [29, 31], suggesting potential additional complexity in the action of SINEUPs.

In previous studies, synthetic SINEUPs have been used to enhance EGFP translation in mouse dopaminergic neuronal cells (MN9D), human cells (HEK293T/17, HepG2 and HeLa) and Chinese Hamster Ovary (CHO) cells. Further, SINEUPs have been used against various targets, including Elastin, various recombinant proteins and Parkinson’s disease-associated DJ-1 protein [9, 10]. *In vivo*, SINEUPs could rescue the phenotypic defects associated with a reduced gene dosage of cox7B in a Medaka fish model of Microphthalmia with Linear Skin Lesions [32]. Accordingly, SINEUPs represent an ideal tool for broader applications in cultured cells (from studying gene function to industrial protein production) and *in vivo* (for treatment of haploinsufficiencies) [33].

To this purpose, we focus on the optimization of the BD and map essential elements of the ED by using synthetic SINEUP-GFP and developing a semi-automated high-throughput screening platform. Here, we explore a variety of BD lengths, and various ED sub-domains in order to develop more powerful synthetic SINEUPs. We identified optimal length and position of BD of synthetic SINEUPs and identified essential elements in the ED, which are crucial for SINEUP effect.

Despite the fact that SINEUP activation involves the formation of dsRNAs in both BD and ED sequences, we proved that SINEUPs do not cause dephosphorylation of 4EBP1 and unwanted dsRNA cellular responses in HEK293T cells system. This is crucial for the use of SINEUPs in therapeutic intervention.

## Materials and methods

### Plasmid and cloning

pcDNA3.1- (Thermo Fisher Scientific) and pEGFP-C2 (Clontech) are commercially available vectors. SINEUP-GFP (FL-60 nt) [5, 9] was used to create all BD and ED mutants. Point mutations were created by QuickChange II Site-Directed Mutagenesis Kit (Agilent) and the mutagenesis PCR primers were designed by QuikChange Primer Design Program (Agilent). ΔBD, SCR-1 and SCR-2 are designed by TransSINE Technologies. BD of SCR-1 and SCR-2 are indicated in below.

SCR-1: ACATCACCCCAAGAAAAGCGGGAACGGTAGCTGGGTCTTGTTAAGATTCCGAGTCTTAACCATCGGAACGAGG

SCR-2:TAGTGCGCCTAAATCGTCAGCAAGATTAGTCATAATCACCTCGGTAGTATCTGTAAAGATCCGCCATAAAAGC

### Cell culture and transfection

HEK293T/17 (human embryonic kidney) cells (CRL-11268, ATCC) and Hepa1-6 (mouse liver hepatoma) cells (CRL-1830, ATCC) were cultured in DMEM (1×) + GlutaMAX-1 (Thermo Fisher Scientific) supplemented with 10% fetal bovine serum (Sigma) and 1% penicillin streptomycin solution (Wako) at 37°C, 5% CO_2_ for 3–5 days. 70–90% confluent cells were treated by 0.05% w/v Trypsin-0.53 mmol/l EDTA 4Na Solution with phenol red (Wako) and 0.5 × 10^6 cells/well were seeded in 6-well plates (FALCON). After 24 h, cells were transfected with pEGFP-C2 and pcDNA3.1-SINEUP-GFP vectors at a molar ratio of 1:4.3 [0.6 μg pEGFP-C2 + 3.6 μg of SINEUP-GFP] with 10 μl of Lipofectamine2000 (Thermo Fisher Scientific). Cells were collected at 24 h post transfection.

### CAGE

CAGE libraries were created using 5 μg RNAs, as previously described [34]. RNA was extracted from HEK293T/17 cells 24 h after transfection of EGFP/SINEUP-GFP plasmids. CAGE tags of EGFP and SINEUP-GFP were sequenced by HiSeq 2000 (Illumina), extracted and analyzed to assess exact TSS position. CAGE sequencing data have been submitted to DNA Data Bank of Japan (http://www.ddbj.nig.ac.jp/index-e.html).

Accession number of Submission: DRA005519

Accession number of BioProject: PRJDB5492

Accession number of BioSample: SAMD00074175-SAMD00074202

Accession number of Experiment: DRX080702-DRX080729

Accession number of Run: DRR086873-DRR086900

### Protein extraction

Cells were washed with D-PBS (-) (Nacalai tesque) and proteins were extracted using 140 μl of Cell Lysis buffer (Cell Signaling Technology) with PMSF (Cell Signaling Technology) per well of a 6-well plate. Samples were slowly rotated at low speed for 1 h at 4°C and then centrifuged (20,000 × g) for 10 min at 4°C. The supernatant was collected and the protein concentrations were measured by DC Protein Assay (BioRad). The absorbance (750 nm) was measured by Multimode Plate Reader ARVO X3 (PerkinElmer).

### Western blot

10–20 μg of extracted proteins were separated by 10% SDS PAGE gel (Mini PROTEAN TGX Precast Gel, 10%, 12-well comb; Bio-Rad) and transferred to a nitrocellulose membrane (Amersham Hybond ECL 0.45 μm; Amersham). Semi-dry transfer was performed using Trans-BLOT SD Semi-dry Transfer Cell (BioRad) with Tris-Glycine buffer containing 20% methanol. The membranes were blocked with 5% nonfat dry milk (Cell Signaling Technology) in Tris Buffered Saline with Tween-20 (Cell Signaling Technology). Proteins were immunoblotted with primary antibodies followed by horseradish peroxidase (HRP) conjugated secondary antibodies. Proteins were detected by ECL Western Blotting Detection Reagent (Amersham) with FUJI LAS-3000 system (FUJIFILM) and FUSION (Vilber-Lourmat). The band intensities were analyzed by Image J version 1.48 software (National Institutes of Health).

### Antibodies

EGFP was detected by anti-GFP rabbit serum A-6455 (Life Technologies), ACTINB was detected by monoclonal anti-β-actin antibody (A5441, Sigma Aldrich), TUBA1A was detected by anti-alpha Tubulin antibody (ab80779, abcam), GAPDH was detected by Anti-GAPDH antibody (G4595, SIGMA Aldrich),PKR was detected by anti-PKR antibody (ab32052, abcam), p-PKR was detected by anti-PKR phospho T451 antibody (ab81303, abcam), eIF2-alpha was detected by anti EIF2S1 antibody (ab26197, abcam), p-eIF2-alpha was detected by anti-EIF2S1 phospho S51 antibody (ab32157, abcam), p-4E-BP1 was detected by anti-phospho-4E-BP1 (ser65) antibody (#9451, Cell Signaling Techonology) and 4E-BP1 was detected by anti-4E-BP1 antibody (#9452, Cell Signaling Technology). Secondary antibodies were horseradish peroxidase (HRP) conjugated Polyclonal Goat Anti-Rabbit antibody (P0448; Dako) and horseradish peroxidase (HRP) conjugated Polyclonal Goat Anti-Mouse antibody (P0447; Dako).

### GFP expression check by Celigo S

A Celigo S Imaging Cytometer (Nexcelom Bioscience) was used for semi-automated high-throughput screening of different SINEUPs. 1.0–1.5×10^6^ cells of HEK293T/17 cells or Hepa1-6 cells were seeded in 24-well Poly-D-Lysine coated plates (Corning). After 24 h, 75 ng of pEGFP-C2 and 725 ng of SINEUP-GFP were transfected with 3 μl of Lipofectamine 2000 (Thermo Fisher Scientific). 24 h post transfection, cells were washed by D-PBS (-) (Nacalai tesque) and the nuclei were stained with Hoechst 33342 (H3570, Thermo Fisher Scientific). The integrated intensity of EGFP was detected and normalized by Hoechst intensity with Celigo S software (Nexcelom Bioscience).

### Total RNA extraction

HEK293T/17 cells were washed with D-PBS (-) (Nacalai tesque) and detached using 0.05% w/v Trypsin (Wako). Cells were collected by centrifugation for 5 mins (6000 × g) at 4°C and total RNAs were subsequently extracted using an RNeasy Mini Kit (QIAGEN) following the manufacturer’s protocol. Samples were treated 3 times with DNase I to digest transfected plasmid DNA with the TURBO DNA-free kit (Thermo Fisher Scientific). RIN values and gel band intensities of the total RNA were checked using the Agilent 2100 Bioanalyzer (Agilent Technologies) following the Agilent RNA 6000 Nano kit protocol. The concentration of total RNA and absorbance at 230/260 and 260/280 were measured by NanoDrop8000 (Thermo Fisher Scientific).

### RT reaction and quantitative RT-PCR

1 μg of the total RNA was reverse transcribed to cDNA using the Prime Script 1st Strand cDNA Synthesis Kit (TaKaRa) following the manufacturer’s protocol. 0.3 μl of random 6 mers primers and 1.7 μl of oligodT primers were mixed to capture RNAs. 1 μl of 10 times diluted cDNA was used as a template for the quantitative real time PCR (qRT-PCR). The qRT-PCR primers were designed with the SYBR Premix Ex Taq (Tli RNaseH Plus) kit (RR420S, TaKaRa). Each reaction contained 1 μl of cDNA, 0.4 μl of reverse primer (10 μM), 0.4 μl of forward primer (10 μM), 10 μl of SYBR pre mix ExTaq, 0.4 μl of ROX reference dye (50 ×), 10 μl of nuclease-free water (reaction volume was 20 μl). EGFP and SINEUP-GFP levels were normalized by GAPDH. qRT-PCR was performed by StepOnePlus Real-Time PCR System (Applied Biosystems) with the following conditions; hold stage (1 cycle) for 30 secs at 95°C, cycling stage (40 cycles) for 5 secs at 95°C and for 30 secs at 60°C, melt curve stage.

RT- samples were performed to check for any plasmid DNA contamination in cDNA. Each sample was performed in technical triplicate (n = 3, Cт standard deviation> 0.2) and with biological replicates (n≥3). 2^-ΔΔCt ±SD was analyzed by StepOne software v2.3 (Applied Biosystems). Primer efficiency was detected as melting curve steps after the PCR stage. EGFP and SINEUP-GFP primers were designed and performed as in the published paper [5], with sequences indicated below:

hGapdh_Fw: TCTCTGCTCCTCCTGTTC

hGapdh_Rv: GCCCAATACGACCAAATCC

EGFP_Fw: GCCCGACAACCACTACCTGAG

EGFP_Rv: CGGCGGTCACGAACTCCAG

SINEUP-GFP_Fw: CTGGTGTGTATTATCTCTTATG

SINEUP-GFP_Rv: CTCCCGAGTCTCTGTAGC

Neomycin resistance_Fw: GCTATGACTGGGCACAACAG

Neomycin resistance_Rv: CCTCGTCCTGCAGTTCATTC

## Results

### BD screening: Sequence requirements for SINEUP-GFP activity and optimized BD design

**Fig 1:**
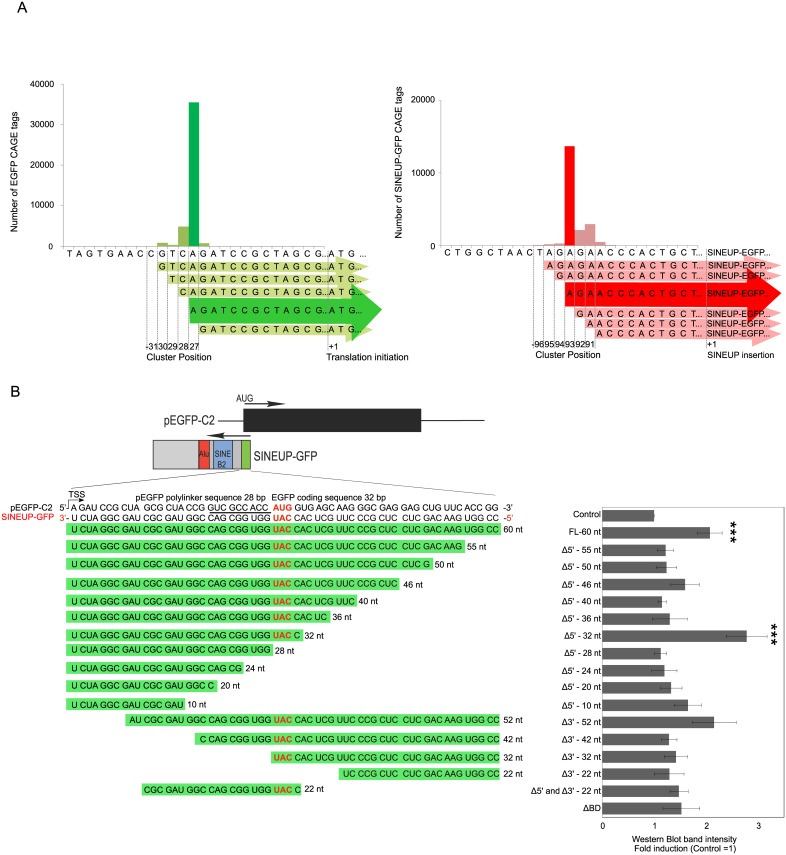
Optimization of SINEUP-GFP binding domain design. (A) TSS analysis of EGFP and SINEUP-GFP by CAGE. CAGE analysis was performed on RNA extracted from HEK293T/17 cells transfected with pEGFP and pcDNA3.1-SINEUP-GFP. Sequencing reads were mapped on reference EGFP (left) and SINEUP-GFP (right) transcripts. Green graph indicates TSS of EGFP and red graph indicates TSS of SINEUP-GFP. The exact position of EGFP and SINEUP-GFP TSS is numbered relative to translation initiation and SINEUP insertion, respectively. (B) Shorter variants of SINEUP-GFP BD show improved activity. Scheme of the anatomy of sense EGFP (derived from pEGFP-C2 plasmid) and SINEUP-GFP transcripts is shown on top. Details of BD sequences used for the screening are indicated. Underlined pEGFP-C2 sequence indicates AUG-Kozak sequence. HEK 293T/17 cells were transfected with pEGFP in combination with SINEUP-GFP or empty control plasmid. EGFP protein quantities were analyzed by Western Blot. Respective EGFP expressions are normalized by ACTINB (endogenous control) fold changes are normalized by control (empty vector). n = 9, ***p < 0.0005, two-tailed Student’s t-test; Error bars are STDEV. Δ: deletion.

We previously designed a synthetic SINEUP against EGFP that successfully acts on EGFP translation [5]. In that study, the BD was designed based on the anatomy of the S/AS overlap of the natural SINEUP AS-Uchl1 [5]. The BD of AS-Uchl-1 is in a -40/+32 configuration, with 40 nucleotides in the 5’ UTR, upstream of the initiating AUG and 32 nucleotides in the coding sequence. Similar BD design was maintained in synthetic SINEUPs targeting exogenous overexpressed genes (GFP, FLAG-tag) and endogenous mRNAs (DJ-1, cox7B) [5, 12, 32]. The exact requirements for BD design to support maximal SINEUP activity are presently unknown. To address this issue, we decided to take advantage of SINEUP-GFP as a model synthetic SINEUP for cell-based assays. Since the BD is designed to cover the initiating AUG and part of the 5’UTR, exact mapping of transcription start sites (TSSs) is required. We investigated cap analysis gene expression (CAGE) libraries [34] using RNA extracted from co-transfected S/AS-GFP plasmids in HEK293T/17 cells. After sequencing with Illumina HiSeq 2500, we mapped the CAGE tags of EGFP and SINEUP-GFP. Clustering of CAGE tags showed that the main peak of pEGFP was 28 nt upstream of AUG (other initiation sites contribute to a minor fraction of the RNAs); the main SINEUP-GFP transcripts start 94 nt upstream of the SINEUP insertion site of pcDNA3.1 (Fig 1A, green is EGFP and red is SINEUP-GFP). By taking advantage of CAGE mapping, we determined the BD of SINEUP-GFP was 60 nt (-28/+32).

To assess whether shorter BDs could further enhance protein translation, we generated a series of antisense variants by deleting several nucleotides from 5’ (Δ5’) and 3’ (Δ3’) BD of SINEUP-GFP (Fig 1B and S1 Fig.). Protein levels were quantified by western blot and all the overlaps were found to have substantial, measurable SINEUP activity. However, this SINEUP activity varies significantly and doesn’t follow a predictable pattern based on the overlap. For instance, one of the SINEUPs (Δ5’-55 nt, 3^rd^ from control in Fig 1B) which constitutes a deletion of 5 bases from the 5’ end of the original 60 nt SINEUP-GFP, reduced protein translation to control levels (Fig 1B). Stronger SINEUPs (Δ5’-32 nt) overlapped the Kozak sequence, “CGCCACCAUGG”, present in the EGFP mRNA, enhancing target protein levels up to 2.8 times, which was higher than the original 60 nt long BD (Fig 1B). These results suggested that the optimal BD of EGFP mRNA should contain the full upstream sequence and should overlap the AUG-Kozak sequence (Fig 1B, AUG-Kozak is the underlined sequence at pEGFP-C2). We next verified whether SINEUP-GFP mutants induce EGFP up-regulation through a post-transcriptional mechanism, in the same manner as the original AS-Uchl1. We found that the levels of the EGFP mRNA did not show statistically significant differences in all conditions (S2 Fig). This result fully supports the translational regulatory mechanism of SINEUPs. Next we tested a BD control plasmid, a BD deletion plasmid (ΔBD) and two scramble sequences of BD (SCR-1 and SCR-2) to check if they show off-target effects. Indeed, ΔBD and SCR plasmids seem to slightly up-regulate translation of GFP, though to a lesser extent than SINEUPs (Δ5’-32 nt) (Panel A in S3 Fig). We wondered whether these plasmids up-regulate translation of other endogenous housekeeping genes. To verify the levels of GFP translation using these plasmids and SINEUPs, we normalized GFP intensities by beta-actin (ACTINB), alpha tubulin (TUBA1A) and GAPDH. Fold induction of ΔBD, SCR and SINEUPs (Δ5’-32 nt) were comparable between the three housekeeping proteins. Although the BD should be designed efficiently and should be compared with several controls, we concluded that the Δ5’-32 nt is the most effective BD (Panels B and C in S3 Fig). The global translation effects, including off-target effects, still remain to be determined by comprehensive combined transcriptional and translational analyses, such as CAGE [35], RNAseq [36] and Ribosome profiling [37] in the future.

**Fig 2:**
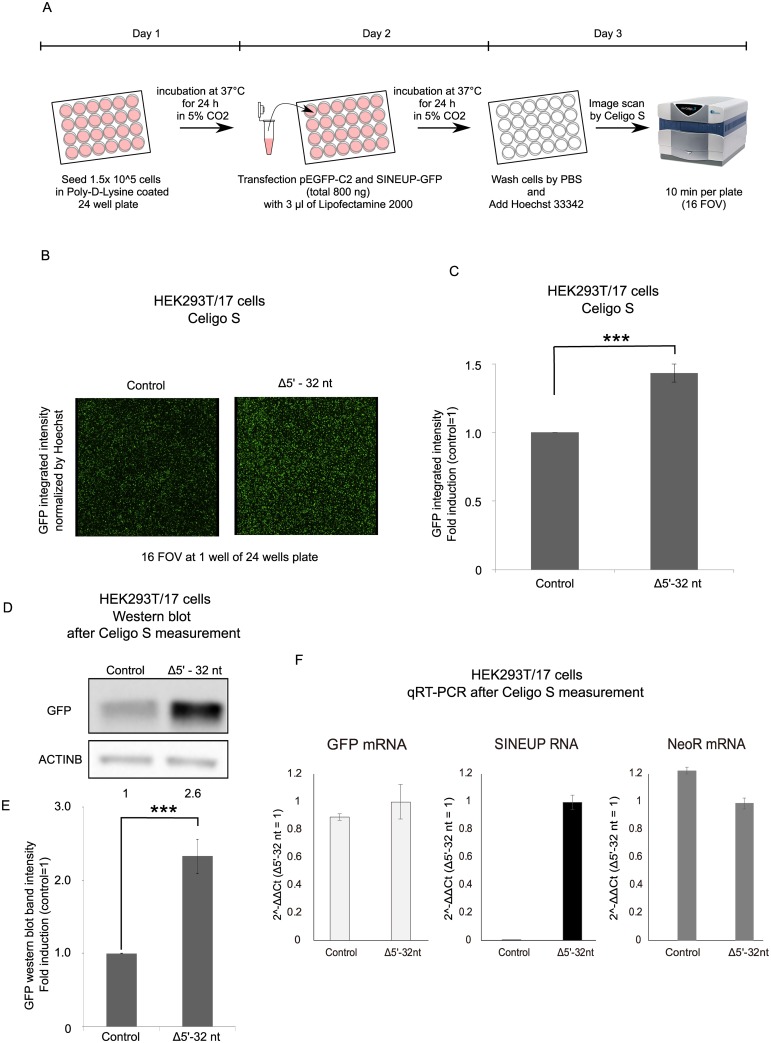
Establishment of semi-automated SINEUP high-throughput detection system by Celigo S. (A) Flow chart of the procedures. (B) Live imaging pictures of EGFP from Celigo S auto-detecting camera. HEK 293T/17 cells were transfected with pEGFP in combination with SINEUP-GFP (Δ5’-32 nt) (right) or empty control plasmid (left). Images are representative of n = 3 independent experiments. (C) Image quantification by Celigo S software. EGFP integrated intensity from cells transfected with control and SINEUP-GFP (Δ5’-32 nt) expressing plasmid. Cell numbers are counted by Hoechst 33342 to normalize integrated intensity. (D) Total proteins were extracted from cells transfected as in B. Proteins were extracted after Celigo S measurement. Western blot analysis was performed with anti-GFP antibody, as indicated. Beta-actin was used as loading control. (E) Quantification of EGFP band intensity normalized to beta-actin in control and SINEUP-GFP (Δ5’-32 nt) expressing cells. (F) GFP mRNA, SINEUP RNA and NeoR mRNA were measured by qRT-PCR. n = 3, ***p<0.0005, two-tailed Student’s t-test; Error bars are STDEV. FOV: field of view.

Given the potential to scale up SINEUP assays, we decided to set up a detection system that would allow semi-automated high-throughput screening of BDs and EDs. pEGFP-C2 and SINEUP-GFP (Δ5’-32 nt BD mutant) were co-transfected into HEK293T/17 cells in 24-well plates, and then living cells were applied to the Celigo S platform (Nexcelom Bioscience LLC) (Fig 2A). Using automated imaging, integrated intensities of the GFP are normalized by total cell numbers, measured by counting the Hoechst-stained nuclei, and are technically controlled during cell seeding steps. As a result, the SINEUPs’ activity was measured as 1.4-fold larger (Fig 2B and 2C). After measurement of SINEUP activity by Celigo S, we prepared cell lysates to accurately measure the SINEUP-mediated increase of target protein levels with western blot. Although Celigo S estimates SINEUP-GFP at 1.4 times, western blot showed a 2.6-fold induction suggesting compression of signals in the Celigo S software calibrated background signals of GFP intensity, in agreement with Fig 1B (Fig 2D and 2E). We further investigated SINEUP-GFP (Δ5’-32 nt) activity in Hepa1-6 (mouse hepatoma) cells by Celigo S platform and western blot proving that SINEUP-GFP (Δ5’-32 nt) exerted its activity also in these cells (S4 Fig). The transfection efficiency of this experiment was measured 1) by normalizing cell number of total cells and GFP positive cells, and 2) by measuring RNA expression of GFP mRNA, SINEUP RNA and Neomycin resistance (NeoR) mRNA that is encoded in pcDNA3.1- plasmid. We confirmed that GFP mRNA expression in the control sample and Δ5’-32 nt sample was almost same and transfection efficiency of co-transfected plasmids, that are measured by NeoR mRNA, was quite similar in control sample and Δ5’-32 nt sample (Fig 2F). In summary, SINEUP activity can be effectively measured by different technologies in different cell lines.

Altogether, we conclude that SINEUP BDs should be designed antisense to the target gene of interest in regions around the AUG and covering upstream untranslated nucleotides.

### ED screening: Sub-domains of the embedded SINE B2 repeat contribute to SINEUP-GFP activity

**Fig 3:**
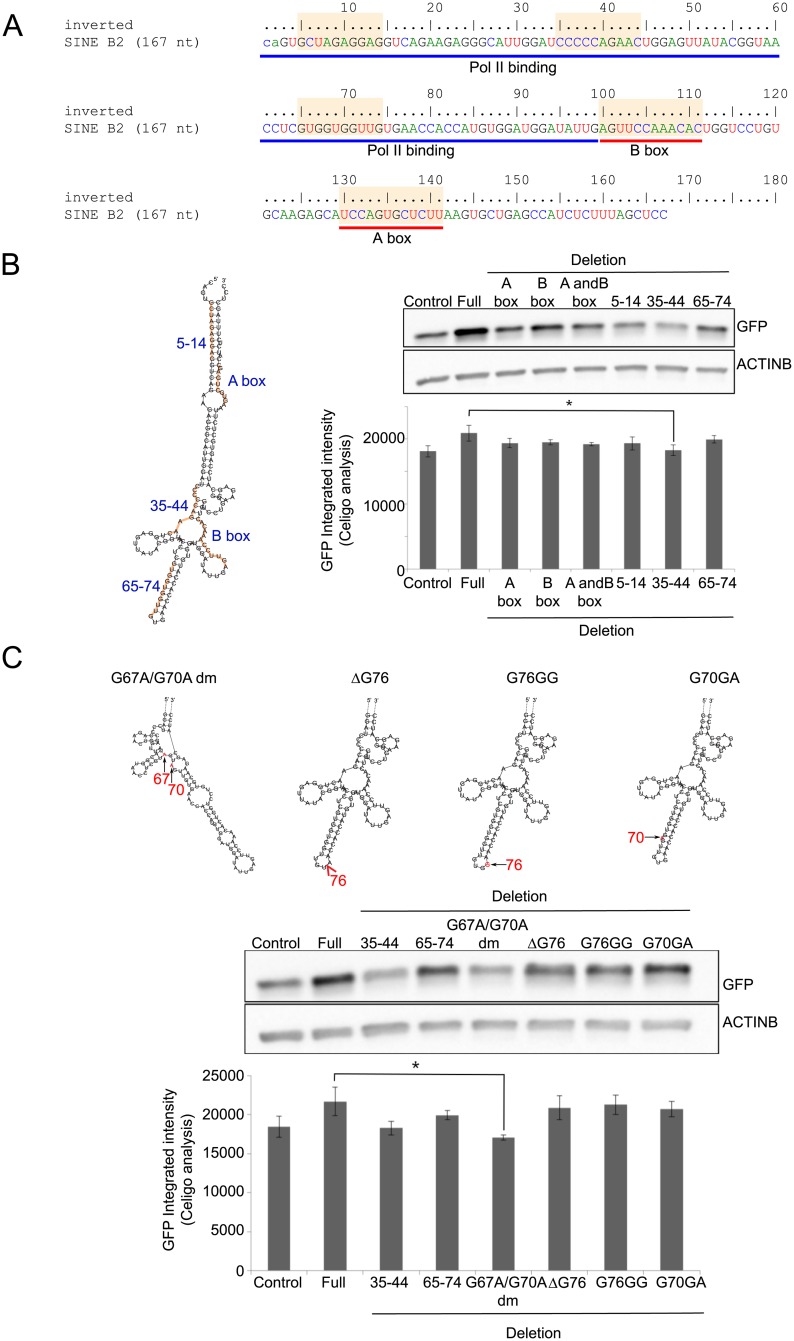
Mapping sub-domains of SINE B2 element required for SINEUP-GFP activity. (A) Sequence and known features of inverted SINE B2 element from AS Uchl1. PolII binding, RNA polymerase II binding region. Orange-marked sequences indicate deletion position of mutagenesis clones for (B). (B) Screening of SINEUP-GFP deletion mutants in sub-domains of inverted SINE B2 ED. Predicted secondary structure of SINE B2 element are determined by RNAfold WebServer (http://rna.tbi.univie.ac.at/cgi-bin/RNAWebSuite/RNAfold.cgi) with default settings (left). Specific sub-domains are highlighted in orange; functional sub-domains and the corresponding nucleotides that are deleted in SINEUP-GFP ED mutants are labelled in blue (see A). All mutagenesis clones are transfected in HEK293T/17 cells with pEGFP-C2 plasmid (right). Western blot results of GFP expressions are normalized to ACTINB and Celigo S results of GFP integrated intensity are normalized by counting whole cell numbers using Hoechst 33342 staining. Activity of ED mutants was compared to empty control (negative control) and SINEUP-GFP Full (positive control). (C) Predicted secondary structure of ED point mutations in the stem-loop region of SINE B2. Activity of SINE B2 point mutants was tested as in B. The 35–44 and 65–74 deletion mutants were included in the analysis. Full: full length SINEUP-EGFP 60 nt, Δ: deletion, dm: double mutation, *p<0.05, two-tailed Student’s t-test, n = 3, Error bars are STDEV.

Our groups and Yao *et al*. previously showed that: (1) the inverted SINE B2 direction is essential for SINEUP to be active [5, 23], and (2) the minimal sequence requirement for the activation are the BD and ED domains (miniSINEUP and miniRNAe) [9, 38]. Since the SINE B2 elements display specific functions associated to their sequence (A box, B box and RNA pol II binding [39] (Fig 3A), we ought to determine if any of these sub-domains contribute to the ED function when embedded in SINEUPs. To address this question, we created a series of deletion mutants (10 nt deletions each) in the ED of SINEUP-GFP (Fig 3A and 3B: orange highlighted sequence). The activity of ED deletion mutants was compared to the FL-60 nt construct upon transfection in HEK 293T/17 cells using Celigo S detection following western blot analysis of GFP protein quantities. Though Celigo S screening results compressed GFP integrated intensity, we confirmed that the deletion of both sequence and structural motifs impaired SINEUP-GFP activity by western blot (Fig 3B). We further wondered if minor sequence changes would be sufficient to abolish the ED function. To test this hypothesis, we mutagenized a few nucleotides at a predicted terminal stem loop region (Fig 3B). Introducing changes to a few nucleotides in this stem loop region (ΔG76, G76GG and G70GA) did not decrease ED activity (Fig 3C). In contrast, changing two guanines (position at 67 and 70) into two adenines significantly decreased SINEUP-GFP activity (Fig 3C). It has to be noted that G67A and G70A mutations dramatically modified the predicted secondary structure in this region. These results suggest that sequence-based and structural-based domains within the SINE B2 element are essential to maintain ED activity in synthetic SINEUPs.

### PKR pathway and dephosphorylation of 4EBP1 are not required for translation activation by synthetic SINEUP-GFP

**Fig 4:**
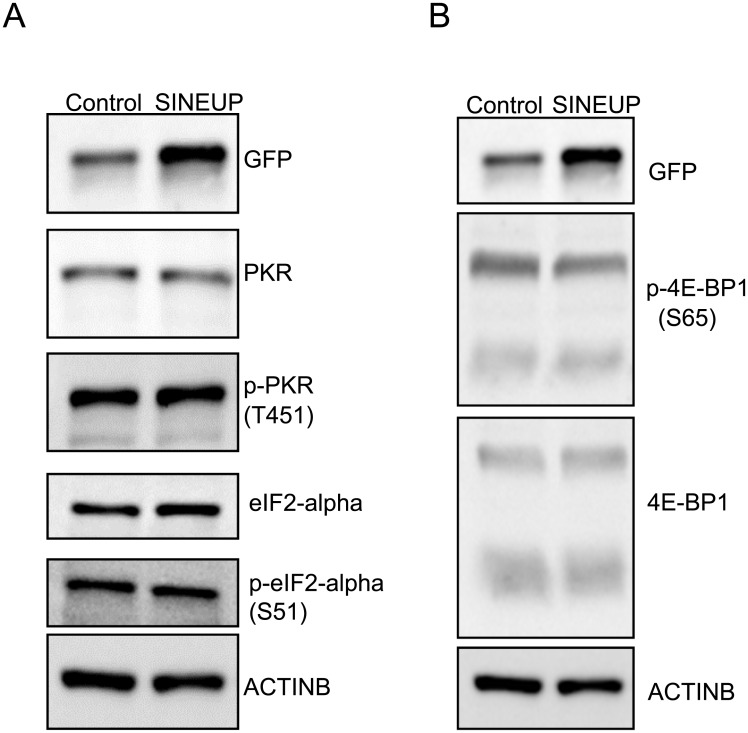
SINEUP-GFP does not alter PKR pathways and 4EBP1 phosphorylation in cultured cells. Effects of SINEUP-GFP on the expression of proteins involved in (A) PKR pathway (B) 4EBP1 phosphorylation.

Since SINEUP BD requires relatively long S/AS overlaps (32 nt to mRNA for the most active one), the resultant large dsRNA duplexes might induce stress responses. We verified if the PKR pathway and interferon activity were activated by SINEUPs. Western blot data shows that the amount of PKR, phospho-PKR (p-PKR T451), eIF2-alpha and phosphorylation of eIF2-alpha did not change after formation of dsRNA hybrid by SINEUP-GFP (Fig 4A). This suggests known translational regulatory pathways are altered when SINEUPs are transfected in culture cells. Activity or phosphorylation of 4EBP1.

We previously reported that Uchl1 mRNA translation is enhanced by the endogenous AS-Uchl1 transcripts upon cellular stress mediated by rapamycin, which inhibits cap-dependent translation and causes dephosphorylation of 4EBP1 in MN9D cells [5]. On the other hand, translation of the exogenous EGFP was not changed by synthetic SINEUP-GFP upon cellular stress by rapamycin and doxorubicin [9]. To compare with the pathway of natural and synthetic SINEUPs, we investigated whether synthetic SINEUPs change phosphorylation of 4EBP1, similarly to the natural SINEUP AS-Uchl1. Western blot analysis showed that overexpression of SINEUP-GFP did not cause noticeable changes in the activity of 4E-BP1, which is known to be associated with cap-independent translation of rapamycin treatment (Fig 4B). Although synthetic SINEUPs show similar function to natural SINEUPs, the translation regulatory pathway of SINEUPs did not change PKR pathway.

## Discussion

Since the SINEUP field is in its infancy, here we have surveyed the sequence features related to BD and ED that control SINEUP activity and examined the possibility that SINEUP BDs may activate double stranded RNA stress response pathways. One of the long-term aims is to understand the rules that govern synthetic SINEUP design in order to widely design SINEUPs “on demand” for mammals and other organisms of interest [9, 10, 12, 32].

Although further studies will be needed on many other mRNA targets varying in length, GC content of UTRs and potential secondary structure, here we have dissected some of the important properties of the BD. In particular, we identified a novel BD (the Δ5’-32 nt), which reproducibly shows high SINEUP activity and requires overlap to the sense AUG and Kozak sequence, such as SINEUP-DJ-1 [9] and SINEUP-NLuc [10]. Further investigations will be needed to understand the mechanisms underlying this BD as the enhancement of translation by an RNA overlapping the AUG and Kozak sequences is counterintuitive. At present, we can speculate that the antisense may reversibly bind to the target mRNA when this is being loaded into polysomes. To achieve high-throughput screening, we have optimized conditions to monitor EGFP translation with western blot and the Celigo S imaging machine, which will enable large-scale screening of EDs and BDs, towards standardization of SINEUP design. More detailed mutagenesis and BD and ED deletion mutants will be needed to further elucidate the underlying mechanism of SINEUP activity. Recent comprehensive RNA secondary structure studies, such as Parallel Analysis of RNA Structure (PARS) in yeast and human, and icSHAPE analysis in mouse embryonic stem cells has revealed that mRNAs often show a single stranded sequence at the end of their 5’UTR and just before the start codon [40–42], supporting the notion that the design of the BD around these regions is likely to be effective. In particular, a relatively shorter region, like in the case of the Δ5’-32 nt construct that fully overlaps both the 5’UTR and start codon may be more effective than larger antisense regions, which may remain partially mismatched or may disrupt other regulatory regions formed by mRNA stem-loops or mRNA-protein interaction.

Additionally, our results support the importance of the inverted SINE B2 RNA secondary structure for translation activation mediated by synthetic SINEUPs. These results fully confirm the data obtained by 2D and 3D structure determination and functional validation of AS Uchl1 (Podbevsšek *et al*., submitted). We hypothesize that when SINEUPs enhance mRNA translation, the secondary structure of the SINE B2 in the SINEUP ED is essential for the recognition of other cellular factors yet to be identified. It is noteworthy that SINE B2 elements shared a common ancestor in their evolution, before diverging into tRNA and SINE B2 [13, 43, 44]. Further studies on the likely complex network of RNA-Protein interactions are needed to decipher detailed mechanisms of SINEUP activity.

RNA therapy promises to address a growing number of genetic diseases. In 1998, Craig Mello and Andrew Fire reported that dsRNA silenced specific genes of *C*. *elegans*, identifying 23-nt-long RNA species that cleaves longer mRNA targets, which opened the field of siRNA [45]. This led to the development of a multitude of approaches aimed at repressing and/or down-regulating target genes involved in the pathophysiology of certain diseases [46, 47]. Importantly, siRNA and similar technologies are very useful for down-regulating target genes [11], yet there are still unmet challenges, such as specific delivery into target cells, RNA stability and interferon activity *in vivo* [48, 49]. SINEUP technology covers a completely different potential treatment niche; by up-regulating protein translation, SINEUPs act in an opposite manner to siRNA technology, thus expanding gene therapy to include haploinsufficiencies, diseases involving an insufficient dosage of target genes [12, 33]. Recently, SINEUPs were shown to correct haploinsufficient gene dosage *in vivo* in a Medaka fish model of human Microphtalmia with Linear skin lesions [32].

Additionally, SINEUPs potentially address one of the concerns related to specificity. In fact, SINEUPs work as a translational enhancer by specifically targeting sense mRNA transcripts only in the cells where they are expressed. Furthermore, SINEUPs are able to increase translation by 2–5 fold [5, 9, 10], thereby SINEUPs may naturally and specifically modulate mRNA translation in diseases caused by haploinsufficiencies. Reassuringly, here we further ascertained that synthetic SINEUP-GFP does not seem to affect PKR pathway activation and cap-dependent translation, which are easing some of the concerns related to future SINEUP applications as RNA therapeutics. Our results here complement previous observations that synthetic SINEUP-GFPs do not require stress, such as rapamycin and doxorubicin, to activate translation of their target [9]. In addition, we demonstrated that synthetic SINEUPs do not require the cap-dependent translation pathway. This is important for their use in restoring physiological conditions of organs and tissues *in vivo*. The next stages of research will involve the delivery of SINEUPs *in vivo*, in particular in rodents. Recently Long *et al*. have produced a transgenic mouse that constitutively expresses a SINEUP to enhance the translation of the mouse growth hormone. This SINEUP (referred to as “RNAe” in their work) caused an increase in body weight [38]. However, detailed quantification of RNA and protein levels *in vivo* and analysis of the molecular networks responsible for the observed phenotype are still needed.

The improved SINEUP design explored in this work will help to develop SINEUPs for therapies, for which there are still many challenges ahead. Among them, the challenge of delivering nucleic acids to target organs is shared with all nucleic acid-based therapies [11]. Side effects of SINEUPs are in principle mitigated by the requirement that the endogenous target mRNA is expressed: the specificity of the BD should ensure SINEUPs only target the endogenous mRNAs present in specific cells and organs. In addition, the optimal required length of the BD is longer than siRNAs, promising fewer non-specific targets. In conclusion, our data show that synthetic SINEUPs consisting of a BD and ED can be determined through a screening system for therapeutic target genes and that said SINEUPs have the potential to be a new and promising tool for RNA-based control of translation.

## Supporting information

S1 Fig SINEUP BD screening. Representative SDS-PAGE images of the western blot results obtained for SINEUP-GFP BD mutants, as described in Fig 1B.
(PDF)

S2 Fig qRT-PCR results for GFP mRNA (black bars) and SINEUP RNA (grey bars) expression. Expression values are normalized to human GAPDH mRNA. Data are analyzed with the ΔΔC_T_ method. FL-60nt mutant is set as 1. Error bars are STDEV. All RNAs were extracted from the same samples shown in Fig 1B.
(PDF)

S3 Fig Non-complementary EGFP target plasmid ΔBD, SCR-1 and SCR-2 slightly up-regulated GFP translation but did not change endogenous translation. (A) HEK 293T/17 cells were transfected with pEGFP in combination with BD control plasmid. GFP protein quantities were analyzed by Western Blot. Respective GFP expressions were normalized by ACTINB (endogenous control), and fold changes are normalized by control (empty vector). (B) and (C) GFP expression were normalized by CTINB, TUBA1A and GAPDH. n = 5, *p < 0.05, **p < 0.005 and ***p < 0.0005, two-tailed Student’s t-test; Error bars are STDEV. Δ: deletion.
(PDF)

S4 Fig SINEUP detection system by Celigo S platform in Hepa1-6 cells. Hepa1-6 cells were transfected with pEGFP in combination with SINEUP-GFP (Δ5’-32 nt) (right) or empty control plasmid (left). (A) 16 FOV of GFP live imaging pictures from Celigo S auto-detecting camera. (B) GFP integrated intensity of control and SINEUP-GFP (Δ5’-32 nt), as calculated by Celigo S software. Cell numbers are counted by Hoechst 33342 to normalize integrated intensity. n = 3, **p<0.005, two-tailed Student’s t-test; Error bars are STDEV. (D) Western blot result of control and SINEUP-GFP (Δ5’-32 nt) activity in Hepa1-6 cells.
(PDF)
