# Supplementary figures and images for: Identification of functional features of synthetic SINEUPs, antisense lncRNAs that specifically enhance protein translation
Source: PLoS One. 2018 Feb 7;13(2):e0183229. doi: 10.1371/journal.pone.0183229 (PMC5802440; doi:10.1371/journal.pone.0183229)

A

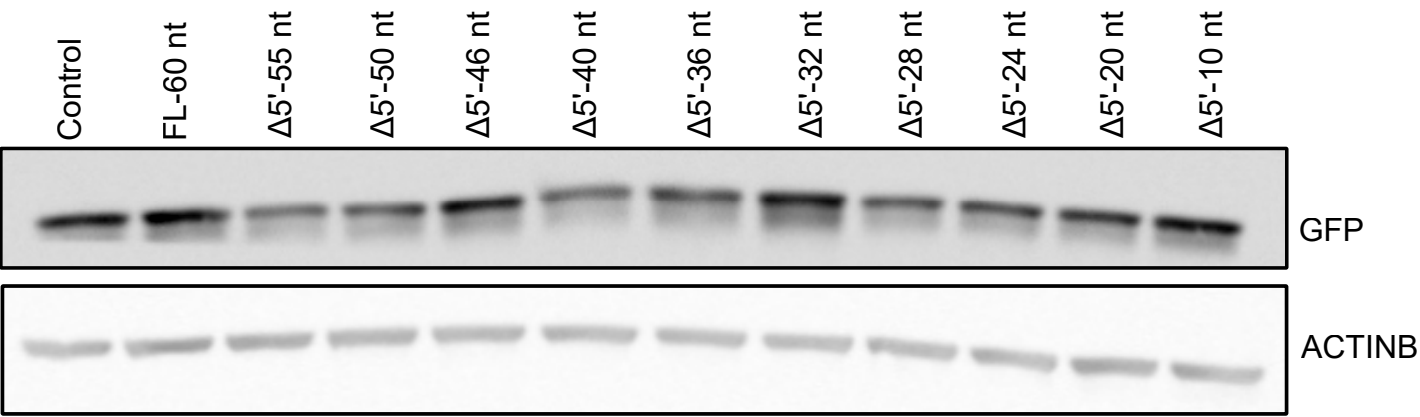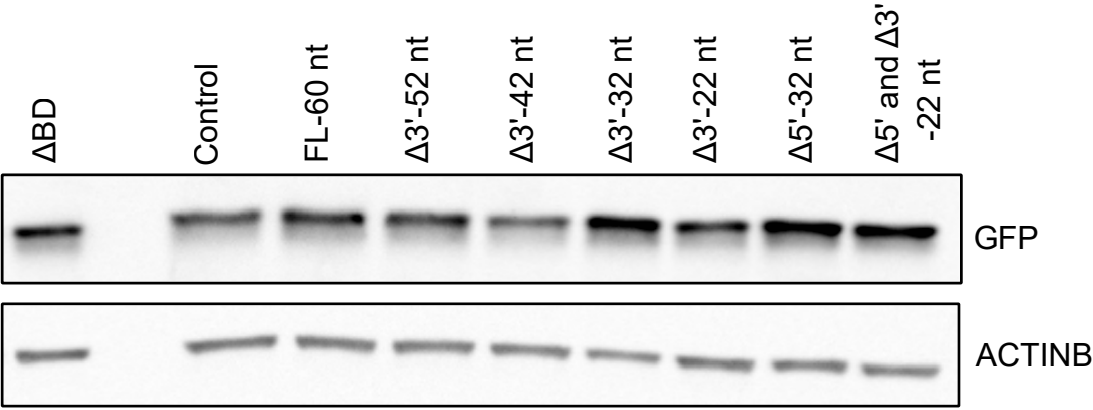

Supplement: S1 Fig — Representative SDS-PAGE images of the western blot results obtained for SINEUP-GFP BD mutants, as described in Fig 1B. (PDF) [file pone.0183229.s001.pdf]

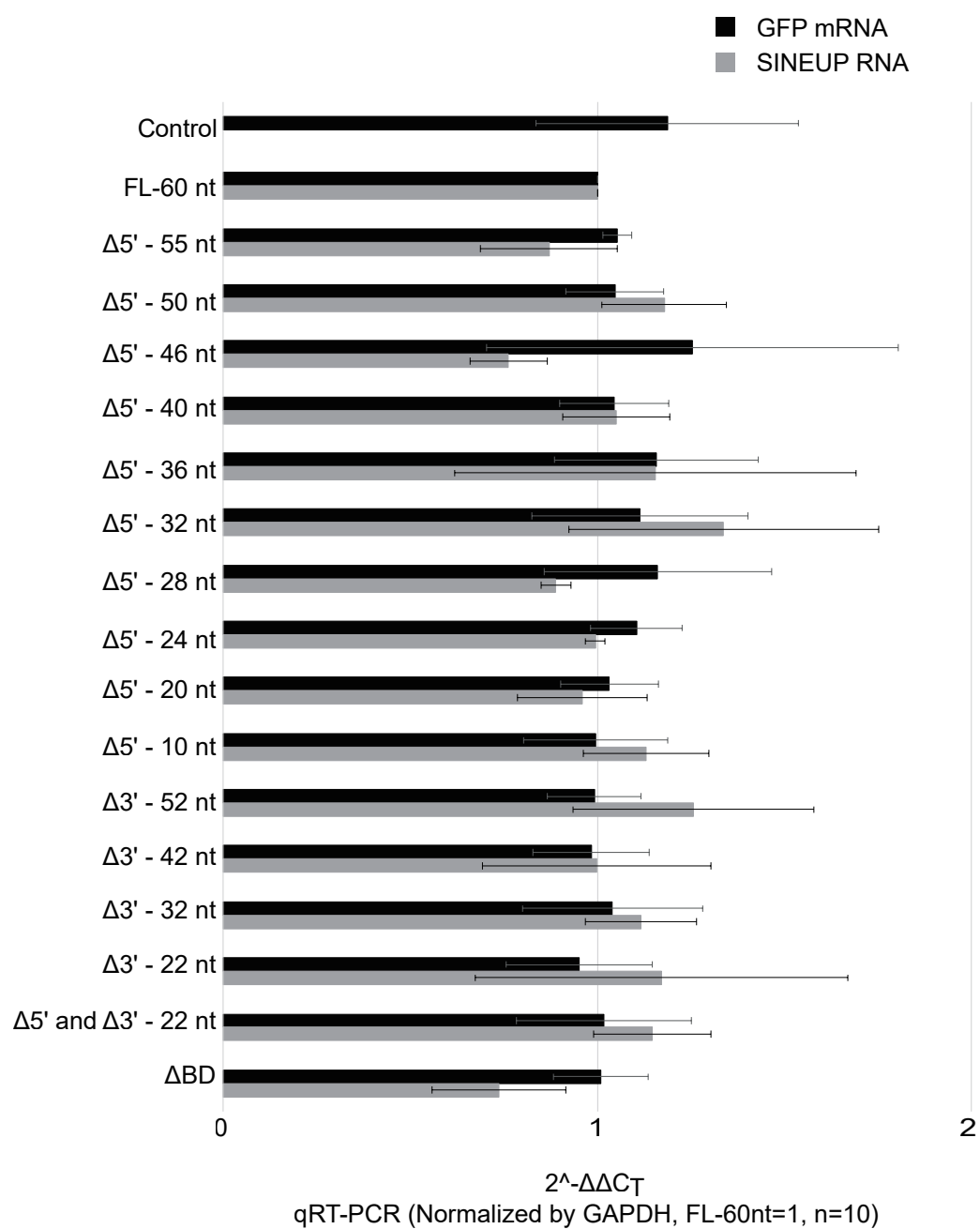

Supplement: S2 Fig — Expression values are normalized to human GAPDH mRNA. Data are analyzed with the ΔΔCT method. FL-60nt mutant is set as 1. Error bars are STDEV. All RNAs were extracted from the same samples shown in Fig 1B. (PDF) [file pone.0183229.s002.pdf]

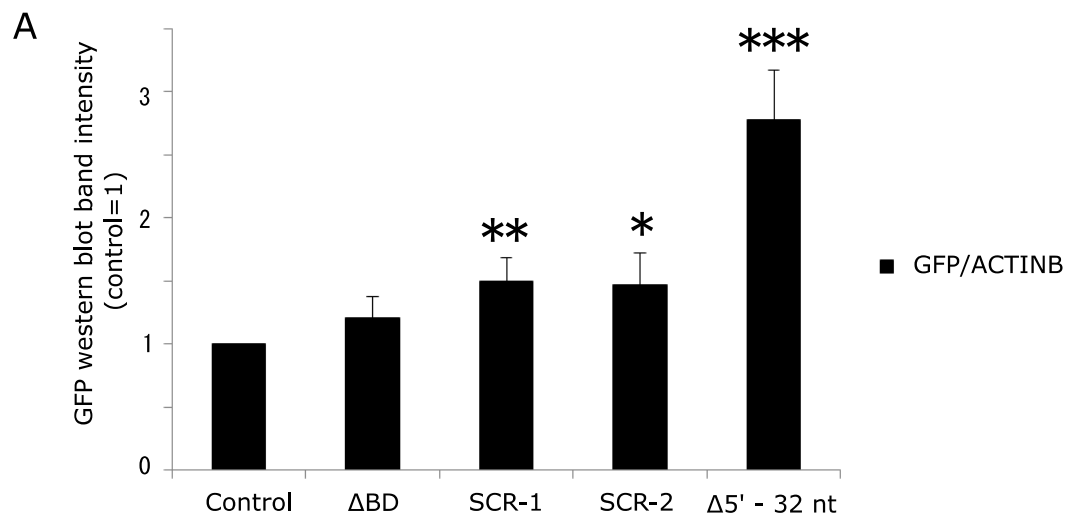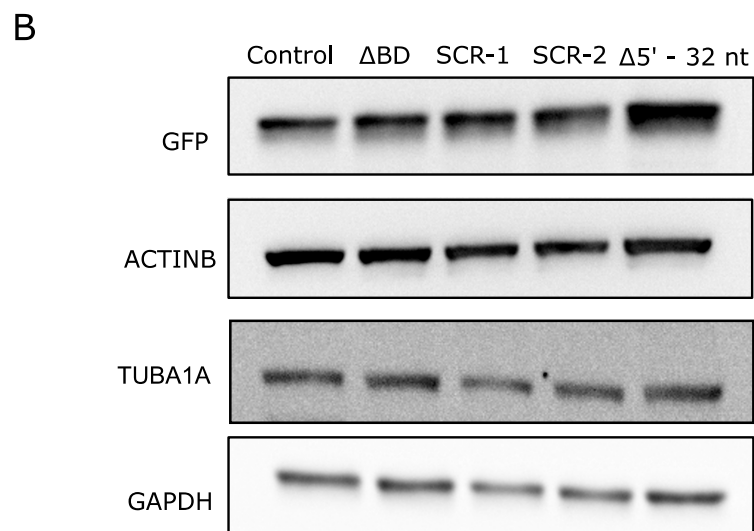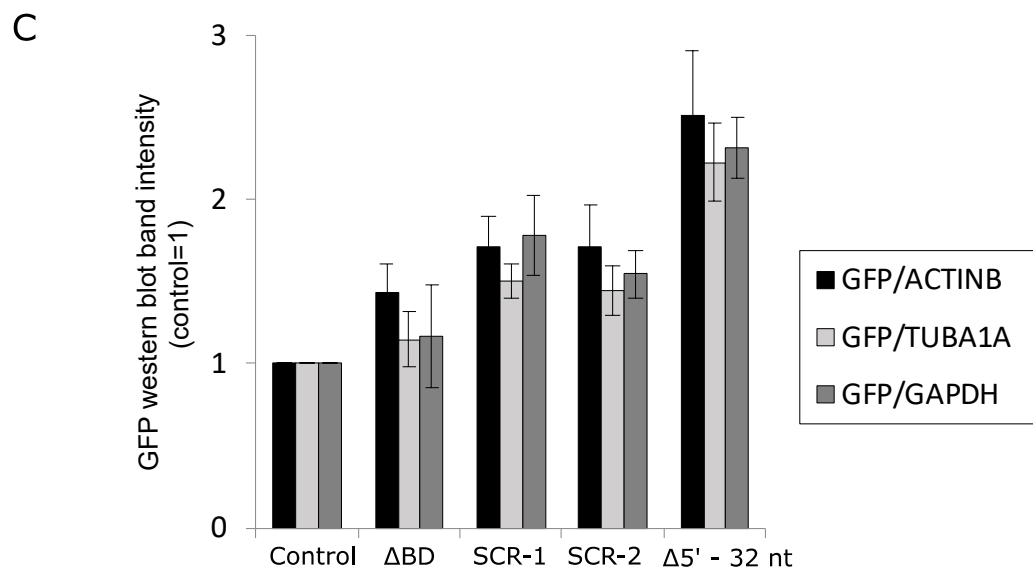

Supplement: S3 Fig — (A) HEK 293T/17 cells were transfected with pEGFP in combination with BD control plasmid. GFP protein quantities were analyzed by Western Blot. Respective GFP expressions were normalized by ACTINB (endogenous control), and fold changes are normalized by control (empty vector). (B) and (C) GFP expression were normalized by CTINB, TUBA1A and GAPDH. n = 5, *p < 0.05, **p < 0.005 and ***p < 0.0005, two-tailed Student’s t-test; Error bars are STDEV. Δ: deletion. (PDF) [file pone.0183229.s003.pdf]

Hepa1-6 cells  
CeligoS

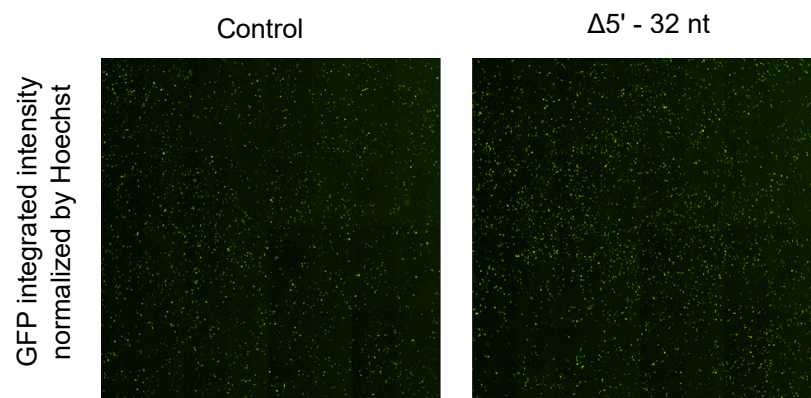

Hepa1-6 cells  
CeligoS

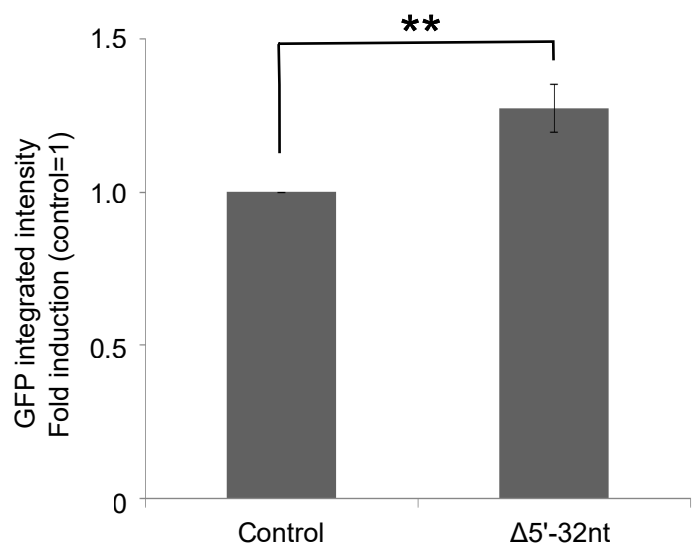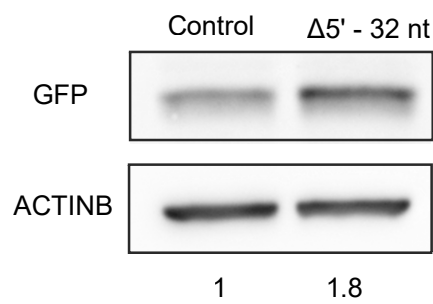

Supplement: S4 Fig — (A) 16 FOV of GFP live imaging pictures from Celigo S auto-detecting camera. (B) GFP integrated intensity of control and SINEUP-GFP (Δ5’-32 nt), as calculated by Celigo S software. Cell numbers are counted by Hoechst 33342 to normalize integrated intensity. n = 3, **p<0.005, two-tailed Student’s t-test; Error bars are STDEV. (D) Western blot result of control and SINEUP-GFP (Δ5’-32 nt) activity in Hepa1-6 cells. (PDF) [file pone.0183229.s004.pdf]
